# Specific Detection of Physiological S129 Phosphorylated α-Synuclein in Tissue Using Proximity Ligation Assay

**DOI:** 10.3233/JPD-213085

**Published:** 2023-03-14

**Authors:** Ryan Arlinghaus, Michiyo Iba, Eliezer Masliah, Mark R. Cookson, Natalie Landeck

**Affiliations:** a Cell Biology and Gene Expression Section, Laboratory of Neurogenetics, National Institute on Aging, NIH, Bethesda, MD, USA; b Molecular Neuropathology Section, Laboratory of Neurogenetics, National Institutes on Aging, NIH, Bethesda, MD, USA; c Division of Neuroscience, National Institute on Aging, NIH, Bethesda, MD, USA

**Keywords:** α-synuclein, phosphorylation, proximity ligation assay, synucleinopathies, immunohistochemistry

## Abstract

**Background:**

Synucleinopathies are a group of neurodegenerative disorders that are pathologically characterized by intracellular aggregates called Lewy bodies. Lewy bodies are primarily composed of α-synuclein (asyn) protein, which is mostly phosphorylated at serine 129 (pS129) when aggregated and therefore used as a marker for pathology. Currently commercial antibodies against pS129 asyn stain aggregates well but in healthy brains cross react with other proteins, thus making it difficult to specifically detect physiological pS129 asyn.

**Objective:**

To develop a staining procedure that detects endogenous and physiological relevant pS129 asyn with high specificity and low background.

**Methods:**

We used the fluorescent and brightfield in situ proximity ligation assay (PLA) to specifically detect pS129 asyn in cell culture, mouse, and human brain sections.

**Results:**

The pS129 asyn PLA specifically stained physiological and soluble pS129 asyn in cell culture, mouse brain sections, and human brain tissue without significant cross-reactivity or background signal. However, this technique was not successful in detecting Lewy bodies in human brain tissue.

**Conclusion:**

We successfully developed a novel PLA method that can, in the future, be used on *in vitro* and *in vivo* samples as a tool to explore and better understand the cellular localization and function of pS129 asyn in health and disease.

## INTRODUCTION

Dementia with Lewy bodies (DLB), Parkinson’s disease, and multiple system atrophy are a group of neurodegenerative diseases termed synucleinopathies. Synucleinopathies are pathologically characterized by the presence of α-synuclein (asyn)-positive protein inclusions in the brain called Lewy bodies (LBs) and Lewy neurites [1, 2]. Prior work has established that the asyn found in LBs is post-translationally modified through nitration, truncation, ubiquitination, SUMOylation, and phosphorylation [3–7]. Of these post-translational modifications, the phosphorylation of asyn at serine 129 (pS129) is the most extensively studied. Under normal cellular conditions pS129 asyn comprises less than 1% of total asyn, whereas about 90% of aggregated asyn is phosphorylated at this residue [3, 8]. Therefore, pS129 asyn has been used as a marker of asyn aggregation and pathology in synucleinopathies and has additionally been explored as a biomarker for disease progression [3, 9–14]. Nonetheless, several aspects of pS129 biology and function remain unclear, including whether phosphorylation of asyn directly influences asyn toxicity, whether it increases or decreases asyn aggregation propensity *in vivo*, or whether it is simply a byproduct of other pathological processes (see review, [15]).

Given the strong ties between pS129 asyn and the pathology of synucleinopathies, it is critical to have tools that specifically detect physiological pS129 asyn. Elucidating the physiologically relevant function of this post-translational modification could allow us to further understand its role in asyn aggregation. Previously developed monoclonal antibodies raised against the pS129 asyn epitope preferentially detect pS129 asyn over non-phosphorylated asyn and readily detect aggregated pS129 asyn but not the soluble form. It has been challenging to limit the cross-reactivity of developed pS129 asyn monoclonal antibodies with other proteins that have similar phosphorylated epitopes, such as neurofilament light chain [16–19]. The significant cross-reactivity of these monoclonal antibodies makes it a challenge to specifically detect and interpret the results from stainings of physiological pS129 asyn. In turn, these challenges have made it difficult to directly study the cellular localization and function of pS129 asyn.

Proximity ligation assay (PLA) is a staining method that creates a robust signal when two primary antibodies are within an estimated range of <40 nm [20]. This range depends on the size of the primary and secondary antibodies, as well as the length of the oligonucleotides attached to the secondary antibodies. When the two conjugated oligonucleotides are close together, they can be ligated and subsequently amplified to create a fluorescent or a 3,3’-Diaminobenzidine (DAB) signal. PLA can therefore be used to demonstrate the close proximity of two proteins within a cell [20, 21]. Additionally, PLA can be used to improve detection of protein modifications by using one antibody targeted to the protein and one targeted to the modification. This approach limits the cross-reactivity and background signal of primary antibodies [22–24]. In the asyn field, PLA has previously been used to stain oligomeric asyn by using a single asyn antibody, and also to detect the association of asyn and α-tubulin in mice and human striatum [25, 26]. Here, we describe a novel PLA system to specifically stain for pS129 asyn using a pS129 asyn antibody MJF-R13 (abcam) and a total-asyn antibody syn-1 (BD Biosciences). We illustrate how this new tool is a sensitive and specific method to detect endogenous and physiological pS129 asyn in cultured cells as well as in WT mouse and human brain tissue. In addition, we show that our novel PLA specifically detects both, physiological and accumulated forms in mouse brain tissue injected with preformed fibrils and mouse brain tissue over-expressing asyn. However, while we could detect overall pS129 asyn in brain tissue from DLB patients, we were unable to directly identify LBs, highlighting the dissimilarities between animal models and human pathology and the limitations of this new tool.

## METHODS

### Primary cultures

Postnatal day 0 WT or SNCA KO cortices were dissected in ice cold HBSS. The cortices were then incubated in papain for 30 min at 37°C. After incubation, cortices were washed twice with BME (Basal Medium Eagle + 0.45% glucose + 0.5 mM glutamate + Pen/Strep + N2 + B27) with centrifugations at 1000 rpm for 3 min. Tissue was triturated in BME containing 50μg/ml of DNAse and then washed twice in BME with centrifugations at 1000 rpm for 6 min. Cells were diluted with BME + 5% fetal bovine serum (FBS) to a concentration of 1.0×10^∧^6 cells/ml. 1.0×10^∧^6 cells were added to each well in a 24-well plate with poly-D-lysine - Laminin coated coverslips. The 24-well plate was then placed in a 37°C and 5% CO_2_ incubator. After 24 h, 80% of BME + 5% FBS was removed and replaced with fresh BME containing 2.5μM Cytosine Arabinoside. Day *In Vitro* (DIV) 6 primary neurons were treated with 1μl adeno-associated virus (AAV6) expressing human asyn or mouse asyn under the synapsin 1 promoter (7×10^∧^13 vg/ml) for 4 h. Cultures were not kept past DIV 9. Primary neurons were fixed by addition of 4 % PFA for 30 min, washed and stored in 1x PBS at 4°C.

### HeLa cultures

HeLa cells were grown in DMEM (Gibco) + 10% FBS (Gibco) and plated on matrigel (Corning) coated glass coverslips at 30,000 cells/well of a 24 well plate. Cells were treated with polo-like kinase (PLK) inhibitor BI2536 at a final concentration of 1μM. DMSO was used as a control. After 4 h, cells were fixed by addition of 4 % PFA for 30 min, washed and stored in 1x PBS at 4°C.

### Animals

Strains used are C57Bl/6J (WT; Jax) and C57Bl/6J 129S6-Snca^tm1nbm^ (SNCA KO) [27]. Mice were housed in a 12-h light/dark cycle with ad libitum access to food and water. All animal work was performed in accordance with the Institutional Animal Care and Use Committee (ACUC) of the National Institute on Aging (NIA/NIH).

### Stereotaxic injection: Viral vectors

Animals (*n* = 4 per group) were anesthetized by 4% isoflurane. After placing the animal into a stereotaxic frame (Kopf), 1μL of AAV6 vector solution (7×10^∧^13 vg/ml) acquired from the NINDS Viral Production Core Facility was unilaterally injected into the substantia nigra (SN) using the following coordinates: anteroposterior (AP) –3.2 mm, mediolateral (ML) –1.5 mm, and dorsoventral (DV) –4.3 mm from bregma. The tooth bar was adjusted to –0.6 mm. Injection was performed using a pulled glass capillary attached onto a 5μl Hamilton syringe with a blunt 22 s gauge needle. After delivery of the viral vector using a pulsed injection of 0.1μl every 15 s, the capillary was held in place for 5 min, retracted 0.1 mm and after 1 min it was slowly withdrawn from the brain. Ketoprofin was administered s.c. as analgesic treatment for 3 days post op.

### Mouse brain sections

Animals were killed when <1 year old or 4 weeks after viral vector injection by an overdose of ketamine and perfused via the ascending aorta first with 10 ml of 0.9% NaCl followed by 50 ml of ice-cold 4% paraformaldehyde (PFA in 0.1 mM phosphate buffer, pH 7.4) for 5 min. Brains were removed and post-fixed in 4% PFA for 24 h and then transferred into 30% sucrose for cryoprotection. The brains were then cut into 30μm thick coronal sections and stored in an antifreeze solution (0.5 mM phosphate buffer, 30% glycerol, 30% ethylene glycol) at –20°C.

### Stereotaxic injection and sections: Preformed fibrils

5μg of asyn preformed fibrils (PFF) (2μg/μl, 2.5μl) was injected to induce α-synucleinopathy [28] into the striatum (anteroposterior (AP) 0.2 mm, mediolateral (ML) 2 mm, and dorsoventral (DV) –3.2 mm from bregma) of wild type mouse, after deeply anesthetized with isoflurane and immobilized in a stereotaxic frame under aseptic conditions. The animal (*n* = 8–10 per group) was observed during and after the surgery and Ketoprofin was administered for three days including the surgery day. Mouse was harvested at 1 month post injection period, perfused with PBS and the brain was fixed with 70% Ethanol in PBS. Then brains were cut into 2-mm thick slices and embedded in paraffin after overnight paraffinizing. The paraffin block was sectioned with 6μm thickness. Before the PLA staining, the slide was deparaffinized with xylene and alcohol series and pretreated with a citrate buffer for antigen retrieval.

### Human brain tissue

Human frontal cortex and hippocampus samples from DLB cases and neurologically unimpaired controls (Table 1) were obtained from the Alzheimer Disease Research Center (ADRC) at the University of California, San Diego (UCSD). Brain tissue was fixed with formalin postmortem and later cut into 40μm thick free floating sections using the vibratome.

**Table 1:** Clinical and demographical data for the patient and control cases. Samples and data were provided by the Alzheimer Disease Research Center (ADRC)

Case ID	Patient ID	Pathology	Secondary Pathology	Date of Death	Age	Sex	Postmortem Hours	BRAAK Stage	MMSE
Case 1	5445	DLB	AD	22 Mar 2009	86	M	12	2	15
Case 2	5421	DLB	AD	16 Nov 2008	86	M	10	1	27
Case 3	5696	DLB	AD	2 Aug 2014	75	M	12	1	20
Case 4	5515	AD changes	N/A	4 Sep 2010	73	F	72	1	30
Case 5	5025	Normal	N/A	2 Jul 1999	51	M	12	0	26
Case 6	5341	Normal	Infarction	20 Dec 2006	77	F	12	0	N/A

AD, Alzheimer’s disease; DLB, dementia with Lewy bodies; MMSE, Mini-Mental State Examination.

### Immunocytochemistry

Neurons were blocked in 5% FBS in PBS for 30 min, and incubated with primary antibodies, diluted 1:500 in 1% FBS/PBS overnight. Following three washes in PBS, the cells were incubated for 1 h with a secondary antibody diluted 1:500 (Alexa Fluor 488-conjugated; ThermoFisher) in 1% FBS/PBS solution. After three PBS washes, the coverslips were mounted and the cells were analyzed by confocal microscopy (Zeiss LSM 880 microscopy).

### Immunohistochemistry: DAB

Endogenous peroxidases were first quenched by incubating sections in 10% Methanol + 3% H_2_O_2_ in PBS for 30 min. Sections were then washed with PBS and incubated for 30 min in blocking buffer (10% Normal Donkey Serum (NDS), 1% BSA, 0.3% Triton in PBS). Afterwards, primary antibodies were used at 1:1,000 and incubated overnight in 1% NDS, 1% BSA, 0.3% Triton in PBS. Next day, sections were washed three times with PBS and incubated with 1:2000 biotinylated secondary antibodies (Vector Laboratories) for 1 h at room temperature. After three washes with PBS, sections were incubated in Avidin-Biotin-HRP Complex solution (ABC kit; Vector Laboratories) for 1 h at room temperature. Sections were then washed three times with PBS and developed using DAB (SigmaFast kit; Sigma-Aldrich). Tissue was dehydrated using increasing alcohol solutions and Xylene, and coverslipped using DPX mounting media. Sections were imaged using a Keyence All-In-One microscope with a 10x or 63x oil lens. Z-stacks were merged using the standard full focus option.

### Immunohistochemistry: Fluorescence

Sections were washed with PBS and incubated for 30 min in blocking buffer (10% Normal Donkey Serum (NDS), 1% BSA, 0.3% Triton in PBS). Afterwards, primary antibodies were used at 1:500 and incubated overnight in 1% NDS, 1% BSA, 0.3% Triton in PBS. Next day, sections were washed three times with PBS and incubated with 1:500 Alexa Fluor 488-conjugated secondary antibody for 1 h at room temperature. After three washes with PBS, sections were mounted on glass slides, coverslipped using Prolong Gold Antifade mounting media (Invitrogen) and imaged using a Zeiss LSM 880 confocal microscope equipped with Plan-Apochromat 63X/1.4 numerical aperture oil-objective (Carl Zeiss AG).

### Antibodies

For a list of antibodies used, please see Table 2.

**Table 2:** Antibodies used to demonstrate cross-reactivity of pS129 asyn monoclonal antibodies and those used for the PLA to specifically detect pS129 asyn are listed below with the provider information and antibody specifics

Antibody	Epitope	Company	Catalog #	Host Species	Clonality	Specific detection of:		
						Human asyn	Rodent asyn	pS129 asyn
Syn1	91-99	BD Biosciences	AB_398107	Mouse	Monoclonal, IgG1	Yes	Yes	No
4B12	103-108	Covance Inc.	SIG-39730-500	Mouse	Monoclonal, IgG1	Yes	No	No
MJF-R13		Abcam	ab168381	Rabbit	Monoclonal, IgG	Yes	Yes	Yes
EP1536Y		Abcam	ab51253	Rabbit	Monoclonal, IgG	Yes	Yes	Yes
81A		Abcam	ab184674	Mouse	Monoclonal, IgG2a	Yes	Yes	Yes
pSyn#64		WAKO	014-20281	Mouse	Monoclonal, IgG1	Yes	Yes	Yes

### Proximity Ligation Assay (PLA)^®^ Sigma Aldrich –Millipore

Reagents. Duolink^®^ *In Situ* PLA^®^ Probe Anti-Rabbit PLUS Affinity purified Donkey anti-Rabbit IgG (H+L) (DUO92002), Duolink^®^ *In Situ* PLA^®^ Probe Anti-Mouse MINUS Affinity purified Donkey anti-Mouse IgG (H+L) (DUO92004), Duolink^®^ *In Situ* Detection Reagents Green (DUO92014), Duolink^®^ *In Situ* Detection Reagents Brightfield (DUO92012), Duolink^®^ *In Situ* Wash Buffers, Fluorescence (DUO82049)

Protocol was generally followed as described by the manufacturer. Briefly, for BF-PLA, sections were incubated in H_2_O_2_ solution for 30 min at room temperature free floating and shaking followed by 2 washing steps with Wash Buffer A. Always ensure that Wash Buffer A is at room temperature before use. All sections were mounted, dried and incubated in Duolink^®^ Blocking buffer for 1 h. All incubation steps were performed in a humidity chamber at 37°C with gentle shaking (50 rpm to ensure an even staining) unless stated otherwise. Afterwards, samples were incubated overnight at room temperature with primary antibodies anti-pS129 (ab209421 MJF-R13, Abcam) and total anti-asyn (42/α-Synuclein syn-1, BD Biosciences) 1:1000 and 1:2000, respectively, diluted in Duolink^®^ Antibody Diluent. The next day, samples were washed 2x for 5 min with gentle shaking using Wash Buffer A. Secondary antibodies Duolink^®^ *In Situ* PLA^®^ Probe Donkey Anti-Mouse IgG (H+L) MINUS and Duolink^®^ *In Situ* PLA^®^ Probe Donkey Anti-Rabbit IgG (H+L) PLUS were diluted 1:5 in Duolink^®^ Antibody Diluent and incubated for 1 h. After 2x 5-min washes with gentle shaking using Wash Buffer A, samples were incubated in 1x Duolink^®^ Ligation Buffer and Ligase diluted 1:40 for 30 min. Then, samples were again washed 2x 5-min using 1x Wash Buffer A with gentle shaking. Samples for F-PLA were incubated for 1.5 h with 1x Duolink^®^ Amplification Buffer (Green) containing the polymerase diluted 1:80. After 2x washes for 10 min with 1x Wash Buffer B and gentle shaking, samples were counter stained with phalloidin-647 (Invitrogen) and DAPI (Invitrogen) for 1 h, washed again using Wash Buffer B, coverslipped with mounting medium (Prolong Gold) and imaged using a Zeiss confocal microscope with an 880 detector and a 63x oil lens. Samples for BF-PLA were incubated for 1.5 h with 1x Duolink^®^ Amplification Buffer (Brightfield) containing the polymerase diluted 1:80. After amplification, tissue was washed 2x 2-min with 1x Wash Buffer A using gentle shaking. Samples were further incubated in 1x Detection Brightfield for 1 h at room temperature. After 2 washes for 2 min with Wash Buffer A and gentle shaking, sections were incubated in Developing Solution containing Substrate Reagent A (1:70), Reagent B (1:100), Reagent C (1:100), and Reagent D (1:50) for 15 min at room temperature. Following 2 washes for 2 min in Wash Buffer A, tissue was dehydrated using increasing alcohol solutions and Xylene, and coverslipped using DPX mounting media. Sections were imaged using a Keyence All-In-One microscope with a 10x or 63x oil lens. Z-stacks were merged using the standard full focus option.

## RESULTS

### Assessment of pS129 α-synuclein antibody specificity using conventional immunostainings

**Fig. 1:**
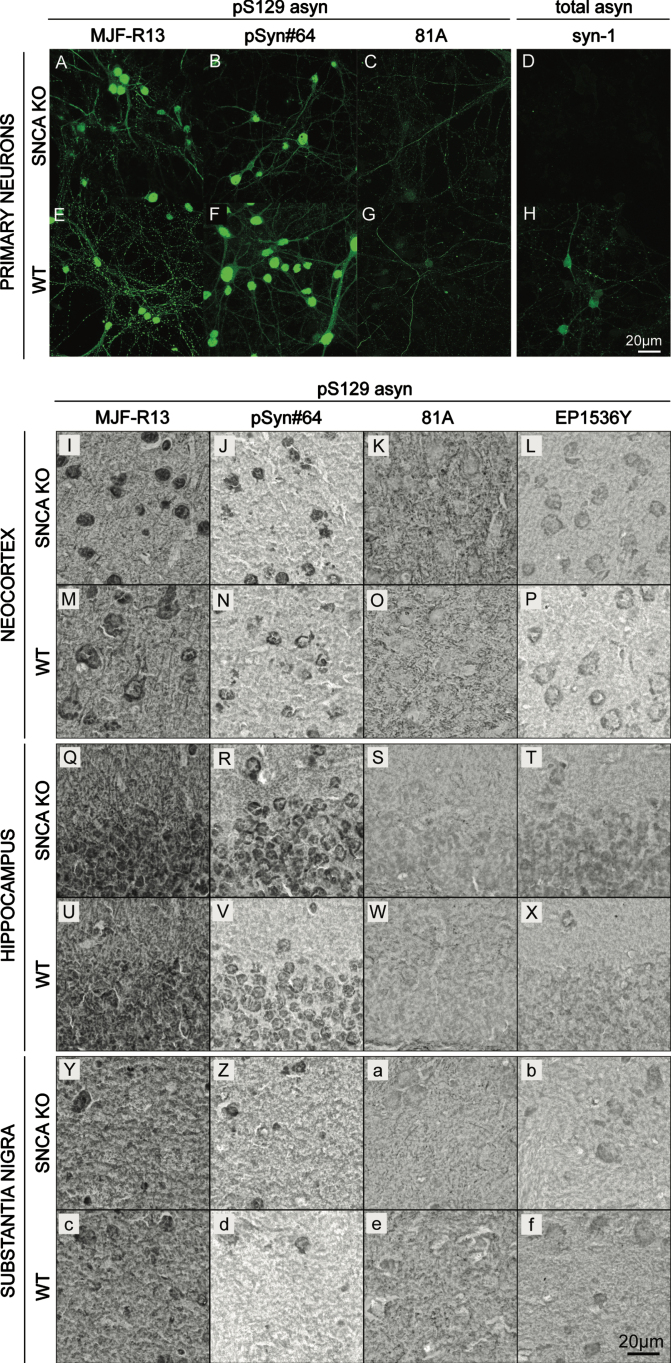
Cross-reactivity of pS129 α-synuclein monoclonal antibodies in cell culture and mouse brain sections. A–H) Immunocytochemistry staining (ICC) of SNCA KO (A–D) and WT (E–H) mouse primary cortical neurons using rabbit MJF-R13 (A,E), mouse pSyn#64 (B,F), and mouse 81A (C,G). Total-asyn antibody, mouse syn-1 (D,H), was used for reference. Alexa-488 stained images were taken at 63x. I-f) Immunohistochemistry staining (IHC) of SNCA KO (I-L,Q-T,Y-b) and WT (M-P,U-X,c-f) 30μm thick coronal mouse brain sections with rabbit MJF-R13 (I,M,Q,U,Y,c), mouse pSyn#64 (J,N,R,V,Z,d), mouse 81A (K,O,S,W,a,e), and rabbit EP1635Y (L,P,T,X,b,f). DAB-stained images were taken at 63x.

To evaluate whether commercially available pS129 asyn antibodies are monospecific, we performed immunocytochemistry (ICC) on mouse primary cortical neurons. We used the pS129 asyn monoclonal antibodies MJF-R13 (abcam), pSyn#64 (WAKO), and 81A (abcam) (Fig. 1 A–C, E–G) [29]. A total-asyn antibody, syn-1 (BD Biosciences), was used as a reference (Fig. 1 D, H) and experiments were performed with both asyn knock-out (SNCA KO; Fig. 1 A–D) and wild-type (WT; Fig. 1 E–H) cultures. MJF-R13, pSyn#64, and 81A antibodies showed extensive staining in both the WT and SNCA KO cultures, demonstrating cross-reactivity of pS129 asyn monoclonal antibodies with other antigens. In contrast, the syn-1 antibody produced a staining in the WT culture only, illustrating that this antibody is monospecific in this application.

Next, we evaluated the specificity of the pS129 asyn monoclonal antibodies MJF-R13, pSyn#64, 81A, and EP1536Y (abcam) using immunohistochemistry (IHC) (Fig. 1 I–f). IHC was performed on paraformaldehyde (PFA) fixed coronal SNCA KO (Fig. 1I–L, Q–T, Y-b) or WT (Fig. 1 M–P, U–X, c–f) 30μm mouse brain sections containing the cortex, hippocampus, and substantia nigra. In all three brain regions, there were no differences in staining patterns between WT and SNCA KO samples. For antibodies MJF-R13, pSyn#64, and EP1536P staining was visible in cell bodies. Additionally, there was a general background staining throughout all brain regions. The 81A antibody did not show cell body staining and had more signal intensity in the cortex and substantia nigra than other areas. In summary, none of the commercially available antibodies that we used here specifically detect physiological levels of pS129 asyn in ICC or IHC.

### Staining of pS129 α-synuclein in cell cultures and mouse brain sections using the proximity ligation assay

**Fig. 2:**
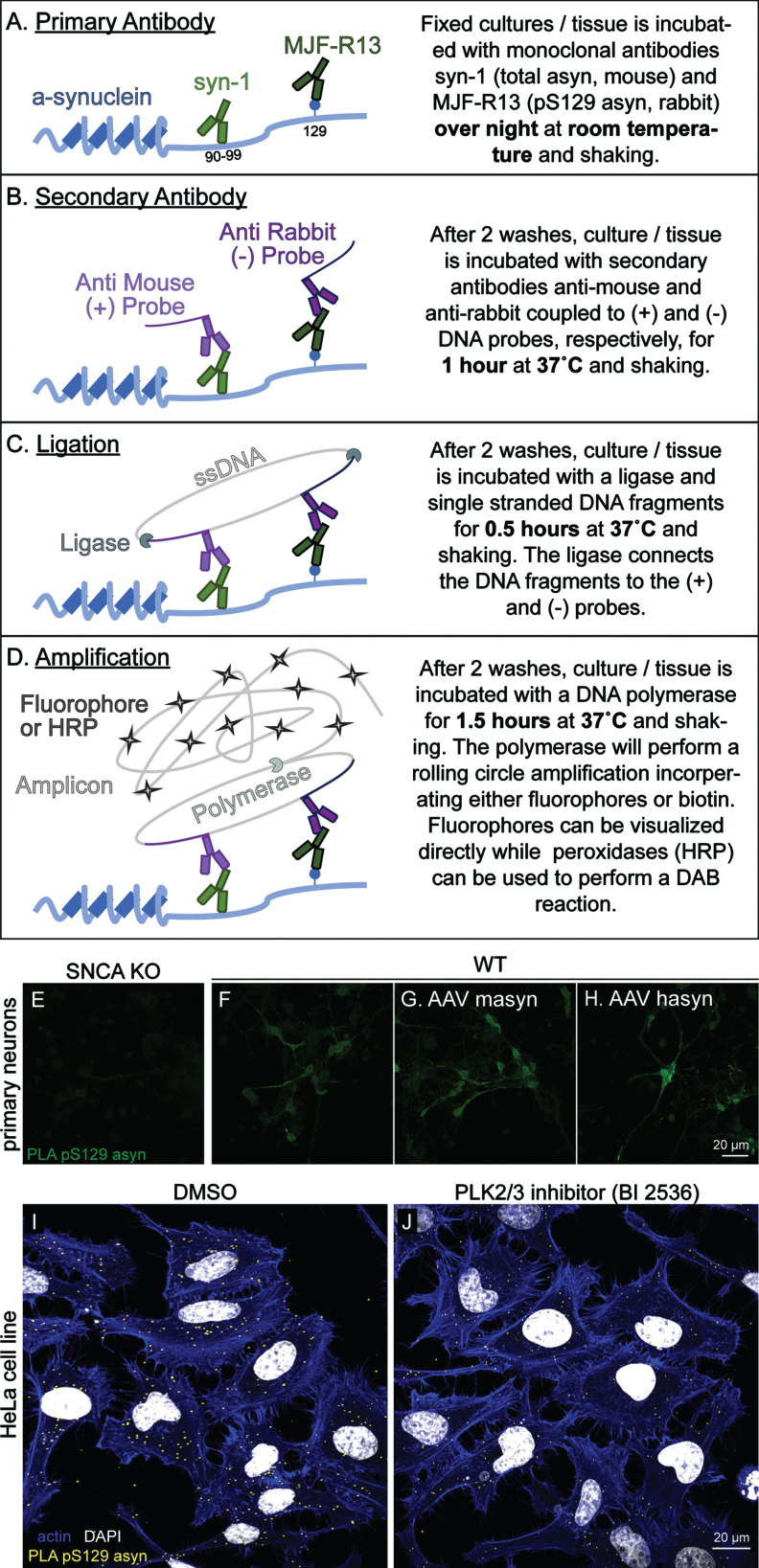
Fluorescent PLA specifically detects pS129 α-synuclein in primary cortical neuron cultures and HeLa cells. A–D) Schematic illustration of the principle experimental steps in the PLA protocol. E–H) The F-PLA specifically stains for pS129 asyn in WT (F) but not in SNCA KO (E) primary cortical neuron cultures. AAV overexpression of mouse asyn (AAV masyn) (G) and human asyn (AAV hasyn) (H) in WT cultures were used to further exemplify the utility of the F-PLA. In HeLa cells, the F-PLA also staines pS129 asyn (I) while cells treated with the PLK inhibitor BI2536 show a reduced staining signal (J).

We next aimed to develop an assay to detect pS129 asyn that would work equally well in rodent and human tissues. Many asyn antibodies that are commercially available recognize the C-terminus of asyn, and typically these antibodies do not recognize both human and rodent asyn with the same affinity due to sequence differences in this region [30, 31]. The syn211 and LB509 clones are human specific monoclonal antibodies that bind very close to the pS129 asyn site [32, 33] and were not considered due to potential competition with pS129 antibodies. The 4B12 and syn204 clones recognize epitopes more distant to S129, but were also eliminated as they do not recognize rodent asyn [8, 32]. We therefore considered C-20 (Santa Cruz) and syn-1 (BD Biosciences) asyn antibodies that can recognize human and mouse total asyn with similar affinity [8, 34]. However, C-20 is no longer commercially available, which led us to choose the mouse monoclonal syn-1. syn-1 is produced in mice and recognizes amino acids 91–99 of asyn (Fig. 2 A) [34]. To achieve a specific PLA signal, we required a monoclonal pS129 asyn antibody with high affinity produced not in mouse to complement the total syn-1 antibody. We therefore chose the rabbit MJF-R13 (R13) clone which has no preference for either rodent or human asyn [8]. An overview of the PLA protocol is shown in Fig. 2 A–D. To evaluate if our PLA specifically stains for pS129 asyn in mouse primary cortical neuron cultures, we performed the fluorescent version of the PLA (F-PLA) in SNCA KO (Fig. 2 E), WT (Fig. 2 F), and WT primary cultures overexpressing mouse asyn (masyn) or human asyn (hasyn) mediated by adeno-associated viral vectors (AAV) (Fig. 2 G and H, respectively). pS129 asyn monoclonal antibody R13 and total asyn monoclonal antibody syn-1 were used for the F-PLA at a concentration of 1:1000 and 1:2000, respectively. Our F-PLA produced a signal in the WT culture that was specific to asyn as demonstrated by the minimal signal detected in the SNCA KO cultures. Signal intensity was further increased in the masyn and hasyn overexpression WT cultures (Fig. 2 G, H). Additionally, we stained HeLa cells with our F-PLA and found that the staining was readily detectable (Fig. 2 I). Using the polo-like kinase inhibitor BI2536 to reduce phosphorylation of asyn on HeLa cells, we could demonstrate a reduction in PLA staining (Fig. 2 J). These data demonstrate that our F-PLA is capable of specifically staining pS129 asyn in primary neuron and HeLa cultures and that it can be used to detect endogenous and physiological levels of pS129 asyn.

**Fig. 3:**
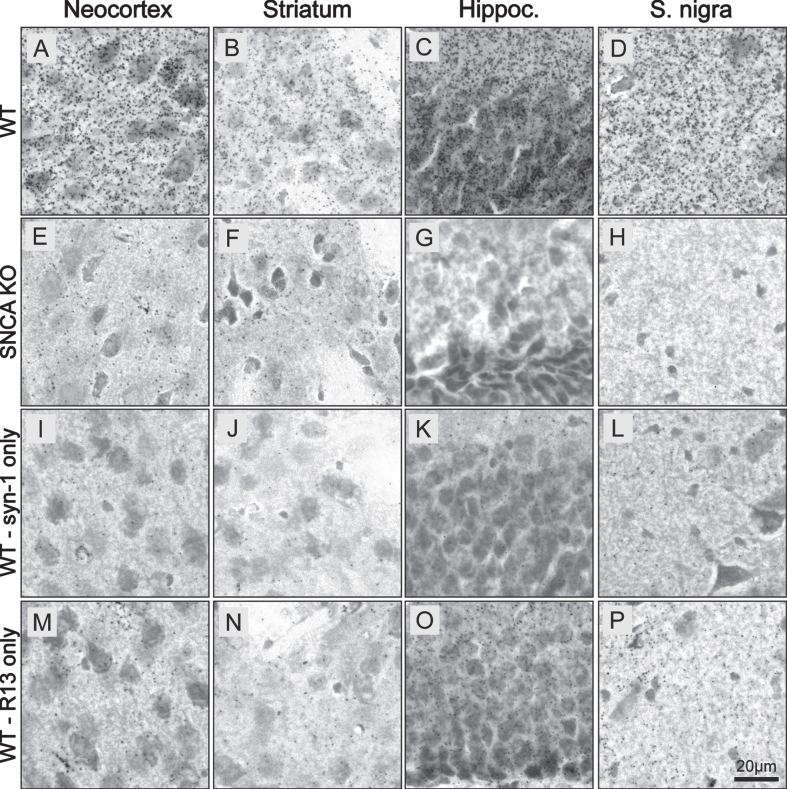
BF-PLA specifically detects pS129 α-synuclein in mouse brain sections. A-P) BF-PLA staining of pS129 asyn was performed on coronal mouse brain sections (30μm). R13 (1:1,000) and syn-1 (1:2,000) monoclonal antibodies were used together (A–H). The BF-PLA was also performed using either syn-1 (I–L) or R13 (M–P) alone as controls. WT (A-D,I-P) or SNCA KO sections (E–H) containing neocortex (A,E,I,M), striatum (B,F,J,N), hippocampus (C,G,K,O), and substantia nigra (D,H,L,P) were used.

We then evaluated the brightfield version of the PLA (BF-PLA) utilizing DAB to stain for physiological pS129 asyn on PFA fixed brain tissue (Fig. 3). pS129 asyn antibody R13 (1:1,000) and total-asyn syn-1 (1:2,000) were again used as the primary antibodies. The BF-PLA was performed with both primary antibodies (Fig. 3 A–H) or with only one of the primary antibodies, either syn-1 (Fig. 3 I–L) or R13 (Fig. 3 M–P) alone. We used WT (Fig. 3 A–D, I–P) and SNCA KO (Fig. 3 E–H) coronal mouse brain sections (30μm) to evaluate the specificity and sensitivity of our pS129 asyn PLA in the neocortex, striatum, hippocampus, and substantia nigra. When both primary antibodies were used, the BF-PLA produced a robust, punctate signal in the WT neocortex, striatum, hippocampus, and substantia nigra (Fig. 3 A–H). The punctate staining is expected as PLA staining relies on rolling circle amplification to produce amplicons that limit diffusion of chromogen from the stained area. Dramatically less signal was produced in the SNCA KO tissue in any of the brain regions (Fig. 3E–H). The syn-1 antibody used alone demonstrated very little nonspecific staining in all brain areas (Fig. 3 I–L) while the sections stained with just R13 alone showed small numbers of punctae, mainly in the hippocampus (Fig. 3 M–P). These findings illustrate that our BF-PLA can be utilized to specifically stain for physiological pS129 asyn in mouse brain sections and that this BF-PLA shows higher specificity than traditional DAB protocols using pS129 asyn monoclonal antibodies.

**Fig. 4:**
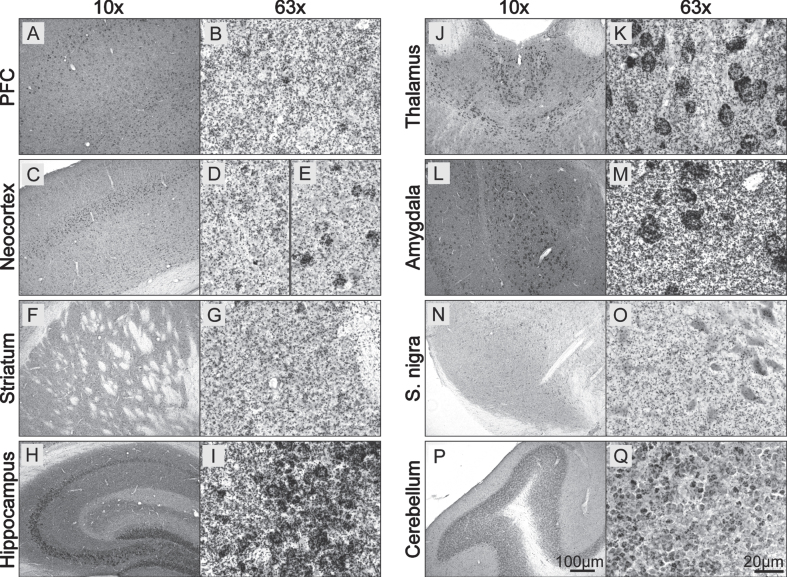
Physiological pS129 α-synuclein staining in the mouse brain. A–Q) WT coronal mouse brain sections (30μm) were stained using the BF-PLA technique. Examples of prefrontal cortex (PFC; A,B), neocortext (C–E), striatum (F,G), hippocampus (H,I), thalamus (J,K), amygdala (L,M), substantia nigra (S. nigra; N,O) and cerebellum (P,Q) are shown in 10x (A,C,F,H,J,L,N,P) and 63x (B,D-E,G,I,K,M,O,Q) magnification.

Next, we evaluated the ability of the BF-PLA to stain for physiological pS129 asyn in additional major brain areas in the WT mouse brain (Fig. 4). A punctate staining was noted throughout the stained mouse brain sections. In the prefrontal cortex (PFC), similar to the neocortex, the staining was not only seen throughout (Fig. 4 A, C) but was particularly strong in layer IV, likely due to staining of neuronal cell bodies (Fig. 4 E). As expected, the pS129 asyn staining was homogenous throughout the striatum and there was no obvious staining of cell bodies (Fig. 4 F, G). The hippocampus was mainly stained in the CA3 region (stratum lucidum) with possible intense labeling of cell bodies (Fig. 4 H, I). Additional somal staining was visible especially in the paraventricular nucleus of the thalamus (Fig. 4 J, K), as well as in the amygdala (Fig. 4 L, M). Even though asyn is well known to be highly expressed in dopaminergic neurons in the substantia nigra and total staining can be visible in their cell bodies (Supplementary Figure 1 W, X), we did not see an increased staining of pS129 asyn in the reticulata or pars compacta subregions (Fig. 4 N, O). Additional intense staining was seen in the granular layer of the cerebellum where round structures, possibly cell bodies were visible with varying strength of staining (Fig. 4 P, Q). These data show that we are able to detect physiological pS129 asyn in primary mouse neurons, HeLas and in mouse brain sections using F-PLA and BF-PLA, respectively. To our knowledge, this is the first time pS129 asyn has been detected at physiological levels in mouse brain tissue without a nonspecific signal. We also demonstrate that pS129 asyn is found throughout the mouse brain and that there is strong pS129 asyn staining in cell bodies in specific brain areas.

### pS129 α-synuclein staining in α-synuclein overexpression animal models and in human brain tissue using BF-PLA

**Fig. 5:**
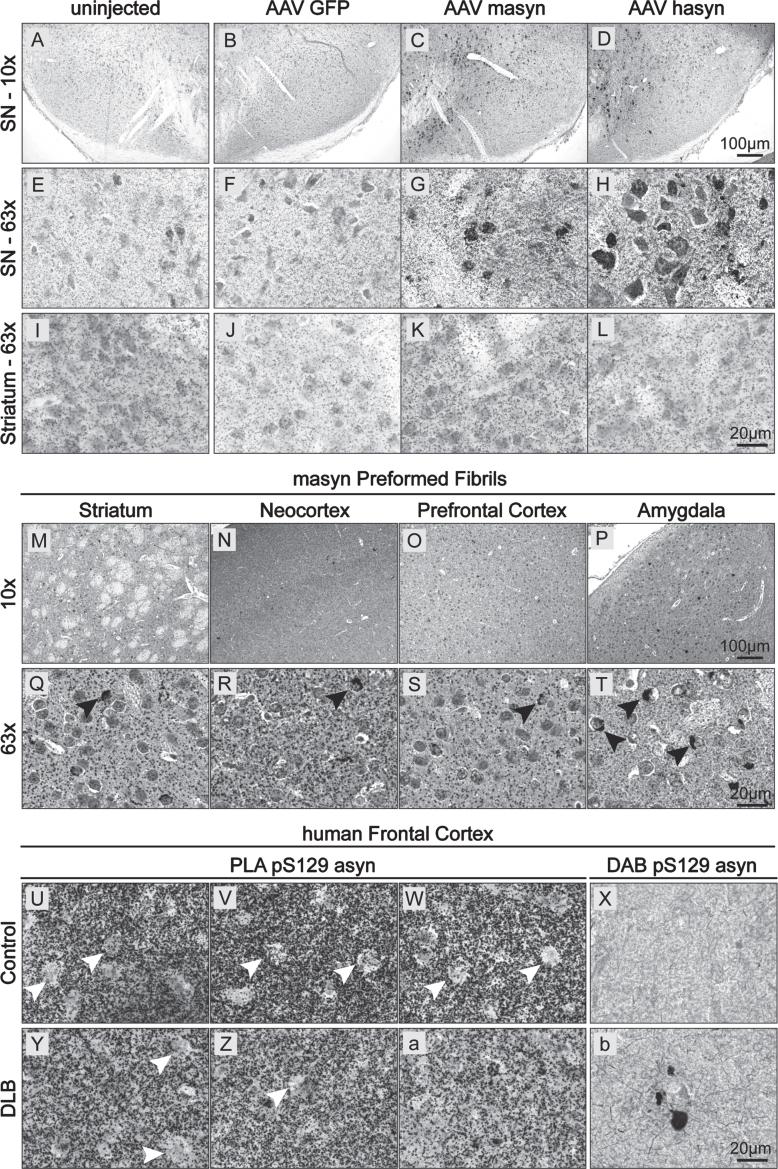
BF-PLA used on sections from animal models and human frontal cortex. A–L) Mice were injected with AAV expressing either GFP (B,F,J), mouse asyn (masyn) (C,G,K) or human asyn (hasyn) (D,H,L). Four weeks after injection, the brains were fixed, cut into 30μm thick sections, and stained using the BF-PLA. Uninjected sides were used as a baseline control (A,E,I). Images were taken either with 10x (A–D) and 63x (E–L) magnification. (M–T) Mice were injected with asyn PFF into the striatum. One month after injection, the mice brains were fixed, embedded in paraffin, cut into 6μm thick sections, and stained using the BF-PLA. Images of striatum (M,Q), neocortex (N,R), prefrontal cortex (O,S) and amygdala (P,T) were taken at 10x (M–P) and 63x (Q–T) magnification. Accumulation of pS129 asyn staining in cell bodies indicated by black arrows (Q–T). U-b) Human frontal cortical sections from a healthy control (Case 5) (U–X) and a DLB patient (Case 1) (Y-b) were acquired from ADRC and stained using the BF-PLA protocol. Images were taken at 63x magnification. The monoclonal pS129 asyn antibody, 81A, was used singly to confirm the presence and absence of LBs in the DLB patient tissue and healthy control tissue, respectively (X,b).

Since pS129 asyn is often used as a marker of asyn pathology, we also wanted to validate our BF-PLA staining of pS129 asyn in relevant animal models (Fig. 5). Detecting soluble pS129 asyn together with accumulated forms can be useful in order to visualize the full picture of pS129 asyn localization, distribution and its changes. This could be particularly of interest when studying pathology in general or when studying the effect of pathology modifying factors. Therefore, we firstly performed BF-PLA staining on mouse brain sections from WT mice overexpressing rodent or human asyn (Fig. 5 A–L). WT mice received unilateral injections into the substantia nigra of AAV6 particles expressing either GFP (Fig. 5 B, F, J), mouse asyn (masyn; Fig. 5 C, G, K), or human asyn (hasyn; Fig. 5 D, H, L) under the synapsin 1 promoter. Four weeks post-injection, we used BF-PLA to stain for pS129 asyn in the substantia nigra and striatum. Uninjected brain sections of WT mice were used in parallel (Fig. 5 A, E, I). Both the AAV-masyn and AAV-hasyn showed increased staining intensity in the substantia nigra compared to the uninjected and AAV-GFP brain sections (Fig. 5 G, H). The additional pS129 asyn signal was mainly found in round structures, possibly cell bodies, and not in the projections of neurons located in the surrounding tissue or in the striatum (Fig. 5 I–L). No obvious differences were seen between the uninjected and control AAV-GFP stainings (Fig. 5 E, F), suggesting that the increase in staining intensity is not due to the AAV injections but rather due to an increase in asyn expression. We then investigated the BF-PLA staining of pS129 asyn in WT mice that received injections of masyn preformed fibrils (PFF). PFFs were injected into the striatum and animals were euthanized 1 month post injection. In line with previous studies, we were able to detect accumulation of pS129 asyn in cell bodies in the striatum, neocortex, prefrontal cortex, and the amygdala of ethanol-fixed paraffin embedded sections (Fig. 5 M–T), with particularly high signals in the amygdala (Fig. 5 Q–T, black arrows). Additionally, we were also able to visualize endogenous, soluble pS129 asyn throughout the brain.

Next, we evaluated the use of BF-PLA to detect non-aggregated and aggregated pS129 asyn of LBs in human brain tissue. Brain tissues from neurologically unimpaired controls and patients with DLB were acquired from the Alzheimer Disease Research Center (ADRC) at the University of California, San Diego (UCSD). In both the control and DLB human brain tissue, BF-PLA showed extensive pS129 asyn staining in frontal cortical areas (Fig. 5 U–W and Y-a, respectively). The staining was lacking in some areas that could potentially be cell bodies (Fig. 5 U–Z, black arrows). However, further studies will need to be performed to confirm the precise cellular localization of pS129 asyn in human brain tissue. Moreover, we stained additional cases as well as brain areas and did not observe an apparent difference in staining intensity between DLB and control groups (Supplementary Figure 1 A–L). Interestingly, our BF-PLA did not readily detect LBs in the DLB human brain tissues (Fig. 5 Y-a, Supplementary Figure 1 B, F, J, D, H, L). To confirm the presence and absence of LBs in the DLB patient samples and healthy control samples, we used the widely used and accepted 81A antibody for DAB staining of pS129 asyn. pS129 asyn aggregates were readily found in the DLB patient tissue while none were detected in the healthy control (Fig. 5 X,b). We performed additional staining using the total human asyn specific 4B12 (1:2,000 dilution) combined with R13 to address whether steric hindrance of the syn-1 antibody (epitope aa 90–99) prevented it from detecting the tightly packed aggregated pS129 asyn. Of note, the combination of 4B12 and R13 has been successfully used in a previously described assay for pS129 asyn [35] and has proven to be human and asyn specific in our hands (Supplementary Figure 1 O, P). As with the syn-1/R13 BF-PLA, we detected soluble pS129 asyn readily using the 4B12/R13 combination, but we again did not detect any LB inclusions (Supplementary Figure 1 M, N). Taken together, our BF-PLA can be successfully used in animal models to detect accumulated and soluble pS129 asyn. Additionally, the BF-PLA is capable of staining soluble pS129 asyn in human brain tissue. However, the BF-PLA assay was not able to detect densely packed asyn in LBs in the tissue of a human patient with DLB.

## DISCUSSION

Here, we describe a PLA protocol that specifically and sensitively detects endogenous and physiologically relevant pS129 asyn in cell cultures, mouse, and human brain tissue. For the first time, we show here the localization and distribution of non-aggregated pS129 asyn in brains that have no pathological attributes present. We additionally show that this PLA can be used to detect increased levels of pS129 asyn in primary cell cultures and mouse models overexpressing asyn, as well as in PFF injected mice. Moreover, we show that a reduction in normal pS129 asyn levels by means of kinase inhibitor can be readily picked up which is further evidence that our assay produces a specific pS129 asyn signal. Therefore, we believe that this PLA method is a great tool to answer some of the major questions still surrounding pS129 asyn biology. Understanding the physiological function of pS129 asyn could help us discern its role in aggregation and pathology. For example, this PLA staining method could be used in combination with other cell organelle markers, such as TGN38 (trans-Golgi network) or Lamp1 (lysosomes), to identify the localization of pS129 asyn, which could suggest a possible function. Furthermore, several co-expression experiments or drug screens could be performed in combination with this method to understand when and where asyn is phosphorylated. Lastly, one could also investigate how the location of pS129 asyn may change over time, such as changes with age, which is a fundamental risk factor for synucleinopathies.

While using the Sigma-Aldrich Duolink^®^ PLA kit, we noticed that staining variations were seen between different batches of kits. We believe this may be in part due to the low-temperature activated Ligase and Polymerase enzymes used during the protocol, which might make them more vulnerable at room temperature. Therefore, special care needs to be taken when using these enzymes. Vials should always be kept in cryo containers and only as short as possible at room temperature. Additional staining differences were seen depending on the experimental setup. When staining 30μm thick brain sections with the BF-PLA, only a thin upper layer (≈5μm) of the section was successfully stained, especially if the section was already mounted before staining and not free floating. Sections were mostly stained already mounted to glass slides to reduce the amount of reagents needed. Better penetration and staining was seen for 6μm thick ethanol-fixed paraffin embedded sections. These penetration issues could come from the antibodies or enzymes not being able to successfully penetrate deep into the sections. Further optimization could be done by using varying amounts of detergent, different fixative, or antigen retrieval to improve staining thickness. While the protocol for our pS129 asyn PLA is more time intensive and expensive compared to traditional ICC or IHC and uses two antibodies produced in mouse and rabbit, it is still possible to co-stain other antigens on the same tissue. In brain sections stained with BF-PLA, immuno-alkaline phosphatases and fast blue can be used as long as the primary antibody is not produced in mice or rabbits. Likewise, F-PLA stainings on sections or cultured cells only require one fluorophore and thus other antigens can be stained if the antibodies are not hosted in mouse or rabbit. Additionally, both F-PLA and BF-PLA are easily interchangeable as the protocols only differ by an additional 2 steps at the end for the BF-PLA (see Method section). The 1st step adds a peroxidase instead of a fluorophore to the amplicon while the second uses DAB to visualize the staining. Therefore, brain sections can easily be stained either with DAB or a fluorophore, making this assay a very versatile method.

Using this novel PLA method, we show here the distribution of pS129 asyn throughout the mouse brain and its cellular localization *in vivo* without major cross-reactivity of the monoclonal antibodies. Interestingly, in the mouse brain, pS129 asyn can be located as a general signal in most parts of the brain but also as more dense staining specifically in some, but not all, cell bodies. This could either suggest a different function of pS129 asyn in distinct neurons or that phosphorylation of asyn is more prominent in cells that highly express the relevant kinases. It is worth noting that the localization of these somal stainings of pS129 asyn in the various subregions do not necessarily overlap with the localization of pathology seen in synucleinopathies [36–38]. In the human frontal cortex, we saw a slightly different pattern. While there was again a general staining in every part of the frontal cortex section, we did not see a localization of pS129 asyn in most of the soma for the cases used here. Additionally, we saw a general staining increase in human compared to mouse brain sections. Unlike conventional staining methods, the PLA most likely produces one amplicon (dot) for one protein of pS129 asyn. The contrast in signal between species could be explained by an overall hyper-phosphorylation of human asyn [39]. We also saw a contrast in BF-PLA signal on the two sets of control/DLB cases. This staining difference may be due to additional variations in handling of human tissue in general. Different tissue blocks may be treated with different fixation solutions and duration, and storage. Different brain regions could also show variations in pS129 asyn protein levels and therefore staining intensity. An additional study focusing primarily on human sections could be performed to investigate this further. Furthermore, true quantifications of protein levels using immunohistochemistry have in general been tricky as these techniques can be easily influenced by many factors such as fixation, postmortem delay and localization on slides. The PLA method has even more components such as ligation and amplification. Therefore, we recommend using this assay only for assessment of protein localization.

Our PLA using R13 / syn-1 was not able to detect pathologically aggregated pS129 asyn in human brain tissue from DLB patients. As the assay does stain accumulated pS129 asyn in the PFF animal model, we hypothesize that this may be due to the chronically aggregated, dense LBs having a different structure or different post-translational modifications when compared to the short-term aggregated asyn in animal models. The syn-1 antibody binds near the 90th amino acid on asyn, which is part of the motif involved in building the β-sheet structure in asyn aggregates [40] and includes a ubiquitination site at K96 [41]. Both these modifications may thus prevent syn-1 from binding to asyn that is aggregated in LBs but is still able to detect endogenous and soluble human pS129 asyn. To rectify this discrepancy, we performed the BF-PLA using 4B12 for total asyn combined with R13 on human DLB tissue. We again detected soluble but not aggregated pS129 asyn. This could either be because of a sterical hindrance of the 4B12 and syn-1 antibodies binding to LBs or due to R13 detecting only a certain set of post-translational modifications besides pS129 that are only found on soluble but not aggregated asyn in humans. Several studies have found that besides phosphorylation on S129, asyn has various other modifications such as ubiquitination, truncation, nitration, SUMOylation, and phosphorylations, for example at Y125 [4–7, 42]. Any of these modifications may influence the nearby epitopes. Coincidentally, a recent paper by Lashuel and colleagues has evaluated the influence of other phosphorylation sites on pS129 asyn antibody binding for 4 major antibodies, including the R13 clone [43]. For example, the R13 antibody itself shows reduced binding to asyn proteins also phosphorylated at Y125. In the future, our PLA method could be expanded to use two complementary pS129 asyn antibodies in combination with syn-1 in order to detect all pS129 species.

Overall, our pS129 asyn PLA with the R13 and syn-1 monoclonal antibodies shows higher specificity than previous stainings with pS129 asyn antibodies alone and is also capable of detecting the low physiological levels of pS129 asyn in cell culture, mouse, and human tissue.

### Conclusion

The PLA described here addresses the issue of cross-reactivity of pS129 asyn monoclonal antibodies by establishing a specific signal for pS129 asyn with minimal background signal. We show how this PLA protocol can be used to specifically detect physiological, non-aggregated pS129 asyn in cell culture, wild-type mouse brain tissue, and human brain tissue. Furthermore, we demonstrate its use in cell cultures and mouse models to detect increased levels of pS129 asyn. While soluble pS129 asyn was readily detected in human brain tissue, LBs were not stained by our PLA protocol in brain tissue from DLB patients. Due to its diverse utility in specifically detecting endogenous and physiologically relevant pS129 asyn *in vitro* and *in vivo*, our PLA can be utilized to answer major questions still surrounding pS129 asyn biology and its role in the pathology of synucleinopathies.

## Supplementary Material

Supplementary Material
