# Supplementary material for: Specific Detection of Physiological S129 Phosphorylated α-Synuclein in Tissue Using Proximity Ligation Assay
Source: J Parkinsons Dis. 2023 Mar 14;13(2):255–70. doi: 10.3233/JPD-213085 (PMC10041430; doi:10.3233/JPD-213085)
Supplement: Supplementary Material [file jpd-13-jpd213085-s001.pdf]

Supplementary Material

Specific Detection of Physiological S129 Phosphorylated α-Synuclein in Tissue Using Proximity Ligation Assay

Supplementary Figure 1. Verification of human cortical BF-PLA staining. Three additional human DLB cases (B,F,J,D,H,L) and three healthy controls (A,E,I,C,G,K) were stained using the BF-PLA (A-L). Sections from the Midfrontal Cortex (A,B,E,F,I,J) and Hippocampus (C,D,G,H,K,L) were chosen. BF-PLA was also performed on frontal cortical human (M,N) and neocortical mouse sections (negative control; O,P) using the human-specific total asyn antibody 4B12 and the previously used pS129 asyn antibody R13. Traditional single antibody DAB staining was used to evaluate the ability of syn-1 (Q,T), 4B12 (R,U) and 81A (S,V) to detect LB in frontal cortical tissue of healthy control (Q-S) and DLB cases (T-V). Mouse nigral sections were stained using the fluorescent IHC protocol for asyn (syn-1; W) and Tyrosine Hydroxylase (TH; X) to identify dopaminergic neurons. All images are taken at 63x using either the Keyence All-In-One microscope (A-V) or the Zeiss LSM 880 confocal microscope (W,X).

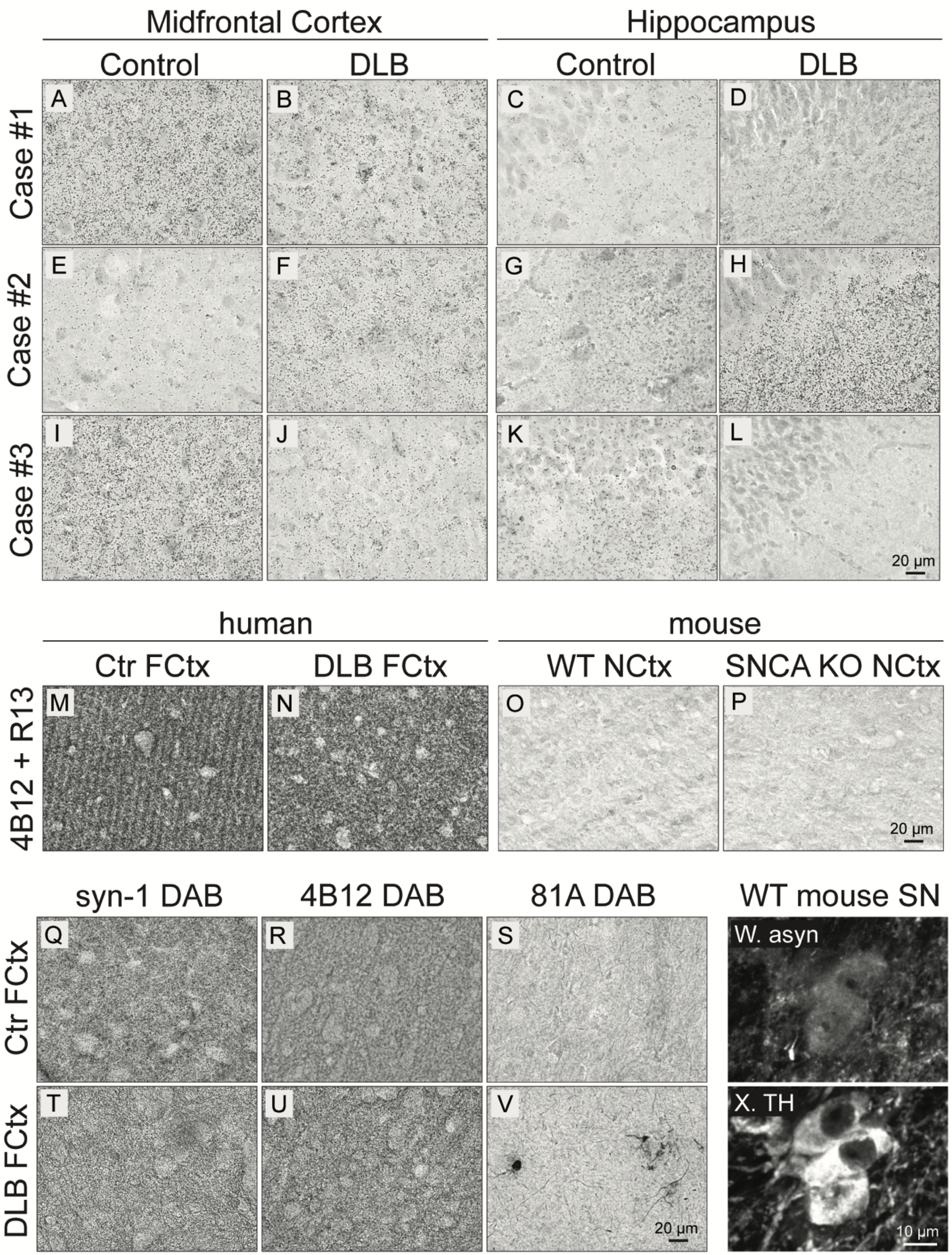

Midfrontal Cortex
Hippocampus
Control
DLB
Control
DLB
Case #1
Case #2
Case #3
A
B
C
D
E
F
G
H
I
J
K
L
20 µm
human
mouse
Ctr FCtx
DLB FCtx
WT NCtx
SNCA KO NCtx
4B12 + R13
M
N
O
P
20 µm
syn-1 DAB
4B12 DAB
81A DAB
WT mouse SN
Ctr FCtx
DLB FCtx
Q
R
S
W. asyn
T
U
V
X. TH
20 µm
10 µm
